# Squamous cell carcinoma of the anus successfully treated with multidisciplinary therapy for metachronous metastatic and local recurrences after DCF chemotherapy: a case report

**DOI:** 10.1186/s40792-024-01873-2

**Published:** 2024-03-25

**Authors:** Ryozan Naito, Takuya Shiraishi, Nobuhiro Hosoi, Takayoshi Watanabe, Ikuma Shioi, Yuta Shibasaki, Nobuhiro Nakazawa, Katsuya Osone, Takuhisa Okada, Akihiko Sano, Makoto Sakai, Hiroomi Ogawa, Makoto Sohda, Ken Shirabe, Hiroshi Saeki

**Affiliations:** Department of General Surgical Science, Gunma University Graduate School of Medicine, 3-39-15, Showa-Machi, Maebashi, Gunma 371-8511 Japan

**Keywords:** Squamous cell carcinoma of the anus, Docetaxel, Cisplatin, 5-Fluorouracil

## Abstract

**Background:**

Docetaxel, cisplatin, and 5-fluorouracil (DCF) chemotherapy is reportedly an effective treatment strategy for squamous cell carcinoma of the anus (SCCA). However, studies regarding its use in Japanese patients remain scarce.

**Case presentation:**

Here, we present the case of an 82-year-old woman with SCCA, cStage IIIB. Chemoradiotherapy was initiated after colostomy of the anorectal mass; however, para-aortic lymph node recurrence was observed 3 months after treatment completion. Five courses of DCF chemotherapy were subsequently administered, resulting in a complete response (CR). Two years and 1 month later, the aortic lymph node was enlarged again, and the patient achieved CR again after radiotherapy. Nine months later, local recurrence was detected in the anal canal, and laparoscopic perineal rectal amputation was performed. The patient remains progression-free 5 years and 10 months after the initial treatment and 1 year and 7 months after the final treatment.

**Conclusions:**

Our findings suggest that complementary treatment after DCF chemotherapy may be efficacious in Japanese patients with SCCA and help achieve CR. Despite occasional local recurrences, this approach may help achieve long-term progression-free survival.

**Supplementary Information:**

The online version contains supplementary material available at 10.1186/s40792-024-01873-2.

## Background

Anal cancer is rare in Japan, accounting for approximately 0.7% of all colorectal cancers [1]. However, its incidence is gradually increasing. Adenocarcinoma is the more common histological type than squamous cell carcinoma in Japan [2]. In contrast, squamous cell carcinoma is more frequent than adenocarcinoma in America, Northern and Western Europe, and Australia. Squamous cell carcinoma of the anus (SCCA) is related to human papillomavirus (HPV) infection [3], and the number of neoplasms associated with HPV-like cervical cancer in Japan is predicted to increase gradually [4]. Therefore, the number of SCCAs in Japan may have increased.

Approximately 80% of SCCA cases are diagnosed at localized or regional stages; typically, SCCA without distant metastases is treated with chemoradiotherapy (CRT) using mitomycin C (MMC) and 5-fluorouracil (5-FU), MMC and capecitabine, or 5-FU and cisplatin (CDDP) [5, 6]. However, disease progression is observed in approximately 30% of cases [7–9]. The recommended management for metastatic or unresectable locally advanced recurrence is chemotherapy with CDDP and 5-FU; however, previous clinical trials failed to achieve a feasible curative effect. The combination of CDDP and 5-FU demonstrated a response rate of 66%, and only 5% of the patients achieved complete response (CR) [10].

The efficacy of docetaxel, cisplatin, and 5-FU (DCF) chemotherapy has been reported in patients with metastatic or unresectable locally advanced recurrences. The Epitopes-HPV02 trial was a study on patients with either SCCA at metastatic stage or with locally advanced recurrence after CRT. It revealed an objective response rate of 89%, and 45% of the patients achieved CR [11]. In this study, 21% of the patients received complementary treatment, and 50% achieved pathological CR. However, there is a lack of international consensus regarding optimal second-line treatment after chemotherapy.

Currently, literature regarding DCF chemotherapy for Japanese patients with SCCA and second-line treatment after DCF chemotherapy remains scarce. Herein, we present a case of double complementary treatment for metachronous metastasis and local recurrence after DCF chemotherapy, in which CR was achieved.

## Case report

**Fig. 1:**
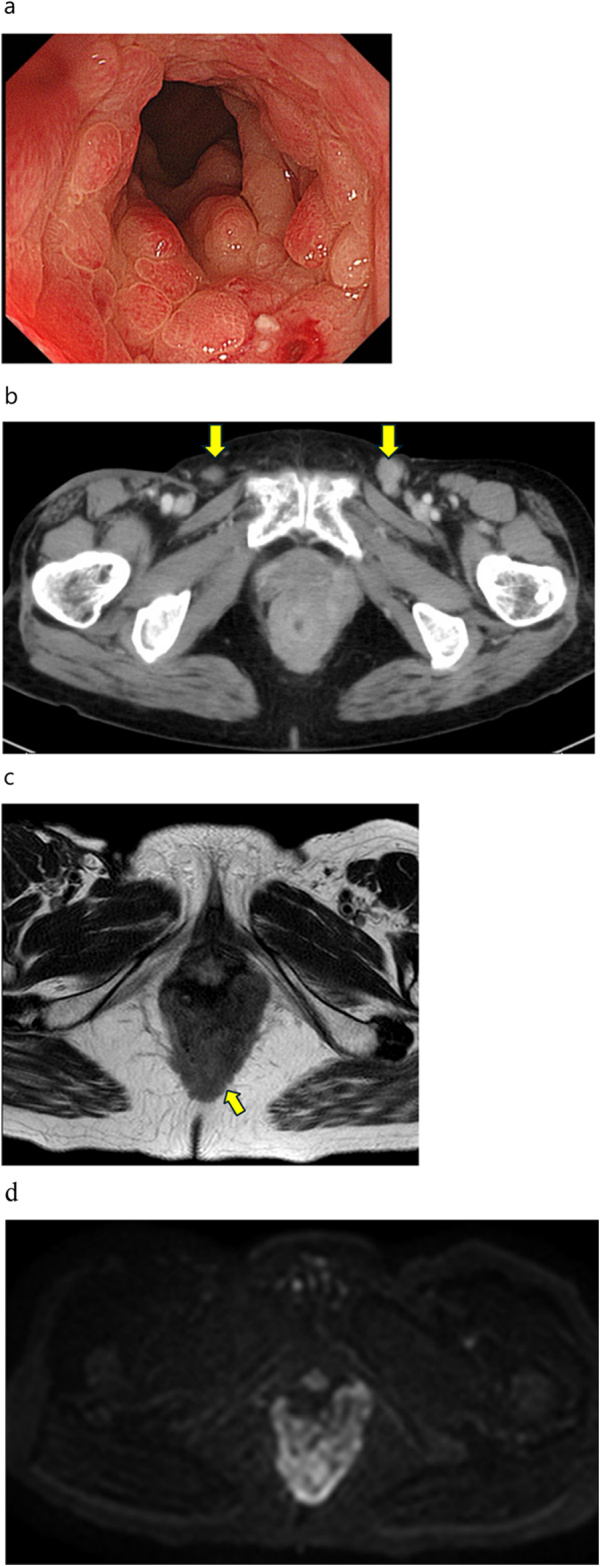
Endoscopic, computed tomography (CT), and magnetic resonance imaging (MRI) findings at diagnosis. **a** Fully circumscribed, indistinct border, type IV tumor in the anal canal. **b** Fully circumscribed wall thickening at the anal canal with metastasis to the bilateral inguinal lymph nodes (arrow). **c** Fully circumscribed wall thickening at the anal canal involving the anal sphincter in T2-weighted image (arrow). **d** The same slice as in **c** in diffusion-weighted image

**Fig. 2:**
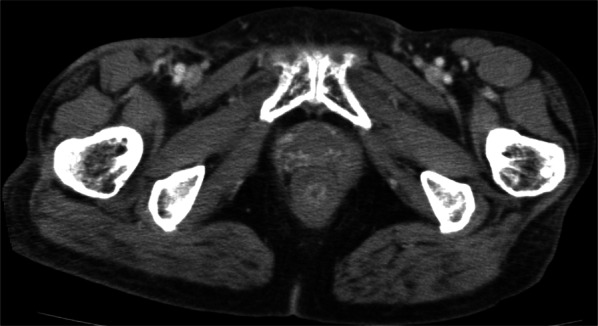
Axial computed tomography slice after chemoradiotherapy. Primary lesion and pelvic and inguinal lymph nodes achieved complete response

An 82-year-old woman presented with a mass in the anorectum that measured > 5 cm and involved the anal sphincter. Endoscopic biopsy revealed moderately differentiated squamous cell carcinoma. Although the tumor had metastasized to the bilateral inguinal lymph nodes, no distant metastases were observed (Fig. 1a, b). The patient was diagnosed with SCCA, cT3N3M0, and cStage IIIB according to the Union for International Cancer Control 8th edition of the TNM classification. After stoma construction, CRT with MMC (10 mg/m^2^ on days 1 and 29), capecitabine (825 mg/m^2^ twice a day on each RT treatment day), and intensity-modulated radiation therapy (IMRT) were administered to the primary tumor and pelvic and inguinal lymph nodes (total dose of 59.4 Gy in 33 fractions). CRT was completed without any major adverse events. Because of the advanced stage, the effect of the treatment was verified as early as 6 weeks after CRT, and the primary lesion and lymph nodes demonstrated CR (Fig. 2). Tumor assessment was carried out according to RECIST criteria version 1.1.

**Fig. 3:**
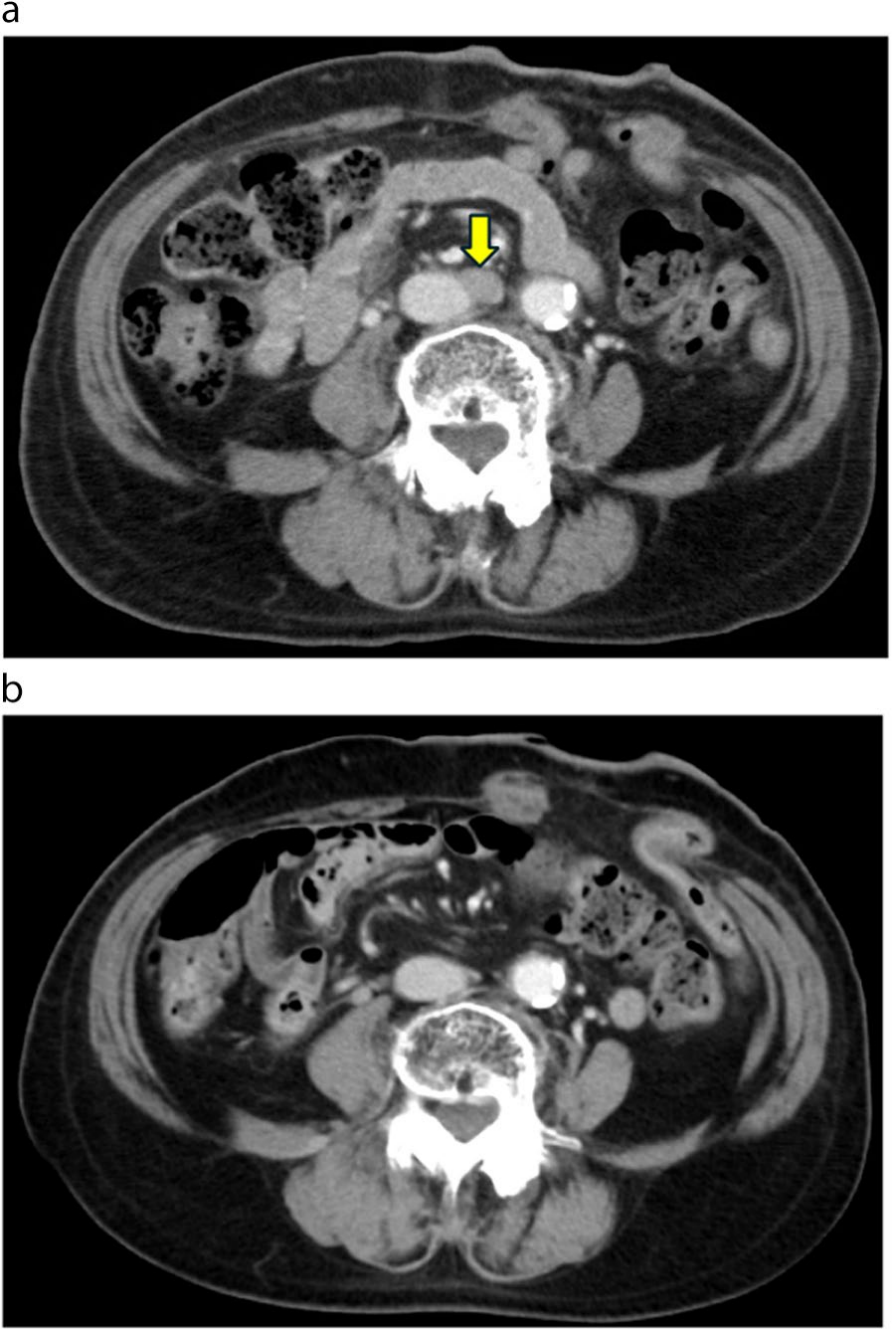
Pre- or post-DCF chemotherapy of the para-aortic lymph node status in an axial CT slice. **a** Para-aortic lymph node recurrence is observed (arrow). **b** After initiating DCF chemotherapy, para-aortic lymph node recurrence demonstrated CR. *DCF* docetaxel, cisplatin, and 5-fluorouracil

**Fig. 4:**
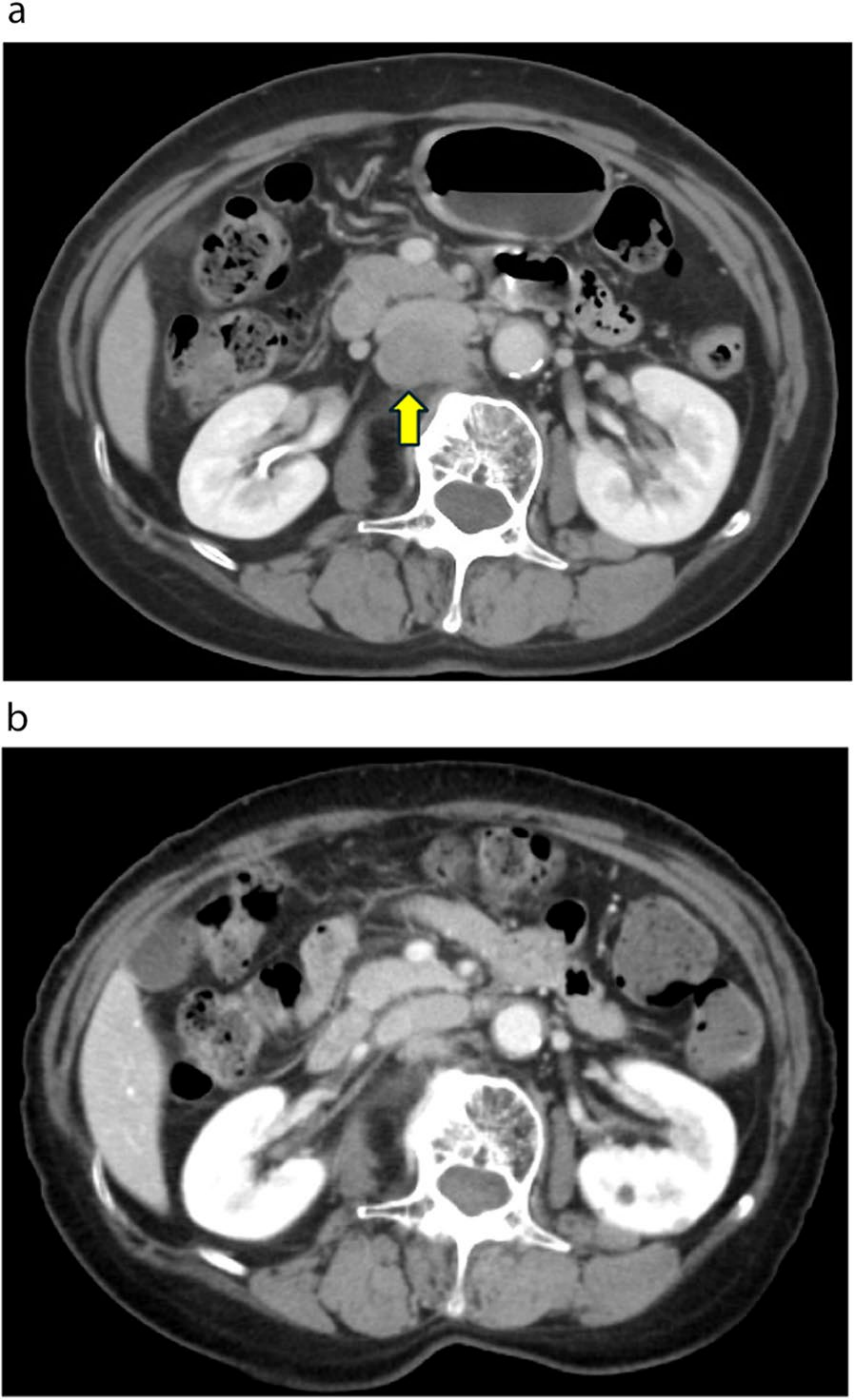
Para-aortic lymph node status pre- and post-radiation therapy assessed using axial CT imaging. **a** Para-aortic lymph node recurrence is observed again (arrow). **b** After intensity-modulated radiation therapy is initiated, para-aortic lymph node recurrence demonstrates CR. *CR* complete response, *CT* computed tomography

Three months after CRT, CT revealed para-aortic lymph node recurrence, whereas CR was maintained in the primary lesion (Fig. 3a). Owing to the advanced age of the patient and concerns regarding side effects, 50%-dose DCF chemotherapy (docetaxel 40 mg/m^2^, day 1; CDDP 40 mg/m^2^, day 1; and 5-FU 400 mg/m^2^, days 1–5; every 3 weeks) was initiated. Treatment was stopped at the fifth course, because febrile neutropenia was observed during the fourth and fifth courses. DCF chemotherapy resulted in CR of the para-aortic lymph node metastases (Fig. 3b). Following DCF chemotherapy, physical examination comprising digital rectal examination, CT, and colonoscopy was performed every 6 months. Stoma closure was not performed because the patient wanted to avoid anal dysfunction. At the follow-up 25 months later, para-aortic lymph node recurrence was observed (Fig. 4a). Because no other recurrences were observed, IMRT (total dose of 60 Gy in 30 fractions for para-aortic lymph node recurrence) was initiated, ultimately achieving CR (Fig. 4b).

**Fig. 5:**
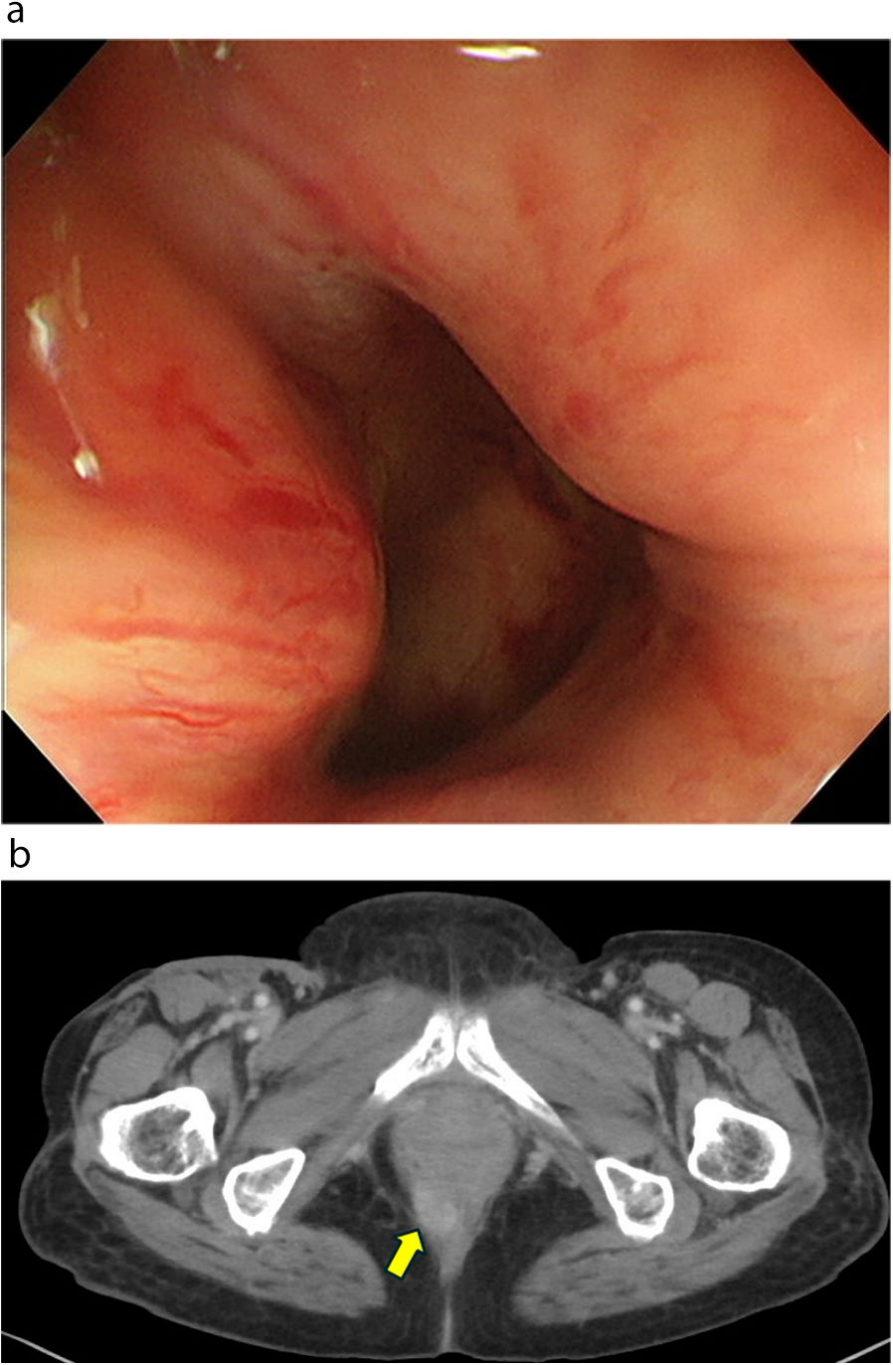
Endoscopic and CT findings at local recurrence. **a** Colonoscopy reveals extramural displacement in the anus. **b** CT image indicating local recurrence of a primary lesion of the anus (arrow). *CT* computed tomography

Nine months after IMRT, local recurrence of the primary lesion of the anus was observed (Fig. 5a and b). The recurrent tumor was located in the region that underwent initial radiotherapy and was exposed to high doses of radiation. Therefore, we performed an abdominoperineal resection, and the postoperative course was uneventful. The patient achieved progression-free survival (PFS) for 1 year and 7 months after surgery, and to date, the patient has survived for 5 years and 10 months after the initial round of CRT.

## Discussion

Herein, we report a case wherein multidisciplinary therapy was applied to successfully manage metastatic and locally recurrent SCCA. The treatment regimen included DCF chemotherapy for metastatic recurrence in the para-aortic lymph nodes following CRT, IMRT for metastatic re-recurrence in the same lymph nodes after DCF, and abdominoperineal resection for the local recurrence of the primary lesion. This comprehensive treatment approach led to complete remission and long-term overall survival (OS) and PFS.

Traditionally, the standard chemotherapy regimen for metastatic SCCA involves CDDP + 5-FU, based on a small retrospective trial [10], which shows a 66% response rate with only one patient achieving CR. However, the efficacy of the DCF regimen for unresectable, locally recurrent, or metastatic SCCA was demonstrated in the Epitopes-HPV02 study [11], where 66 patients were treated with standard or modified DCF regimens, resulting in an 89% response rate, with 30 patients achieving CR. Additionally, the InterAct study reported the effectiveness of carboplatin (CBDCA) and paclitaxel (PTX) regimens for inoperable locally recurrent or metastatic diseases [12]. Taxol anticancer agents have been used owing to their association with HPV and SCCA [13]. HPV types 16 and 18 encode the E6 oncoprotein, which degrades the tumor suppressor gene p53 [14]. p53-deficient cells are more sensitive to taxol antitumor agents owing to increased G2/M arrest and apoptosis. Therefore, the addition of docetaxel to the 5-FU and CDDP regimens has shown promise [15]. The 2023 National Comprehensive Cancer Network (NCCN) guidelines recommend these regimens and others for metastatic anal cancer [5], with recent retrospective studies favoring the DCF regimen over doublet regimens (FU + CDDP or CBDCA + PTX) in terms of OS and PFS [16].

In our case, DCF chemotherapy was administered for metastatic recurrence following CRT, and CR was achieved. Subsequently, the limited metastatic recurrence in the para-aortic lymph nodes was treatable using radiotherapy. DCF chemotherapy has demonstrated effectiveness in preventing metastatic recurrence of SCCA. Although HPV testing was not conducted in this case, considering the HPV status when selecting taxol as an antitumor agent is recommended.

The 2023 NCCN guideline recommends surgery for patients with locally recurrent disease and systemic chemotherapy for those with extrapelvic metastatic disease. However, there is no consensus on the optimal second-line treatment, and recommendations for local recurrence after metastatic recurrence, as in this case, are lacking. Analysis of secondary treatment in the Epitopes-HPV01 and Epitopes-HPV02 studies suggests that complementary treatments, such as surgery or radiotherapy, may lead to better prognosis [17]. Fourteen patients received complementary treatment, and their median OS and PFS were 48.3 months (NE-NE) and 31.3 months (23.2–NE), respectively. In contrast, 59 patients received systemic treatment, such as chemotherapy or immunotherapy, and their median OS and PFS were 11 months (8.4–15.4) and 4.9 months (3.3–7), respectively.

In our case, radiotherapy was administered for metastatic recurrence after DCF chemotherapy, and abdominoperineal resection was performed for local recurrence. This approach effectively suppressed long-term distant recurrence by treating para-aortic metastases with radiotherapy after suppressing micrometastases with DCF. Local recurrences of the primary tumor were completely resected without causing distant metastasis. This case highlights the effectiveness of complementary treatments, even for metachronous recurrence.

## Conclusion

We were able to achieve long-term survival with radiotherapy and surgery following DCF therapy for metachronous local and metastatic recurrence of SCCA post-CRT  (Additional file 1). This highlights the favorable efficacy of DCF chemotherapy and complementary treatments as a second-line strategy. In the absence of a consensus on the optimal second-line treatment approach, this case emphasizes the potential effectiveness of complementary treatments following DCF chemotherapy.

### Supplementary Information

**Additional file 1.** Additional images.

## Data Availability

All data generated or analyzed during this study are included in this published article.

## Abbreviations

DCF: Docetaxel, cisplatin, 5-fluorouracil
SCCA: Squamous cell carcinoma of the anus
CR: Complete response
HPV: Human papilloma virus
CRT: Chemoradiotherapy
MMC: Mitomycin C
5-FU: 5-Fluorouracil
CDDP: Cisplatin
IMRT: Intensity-modulated radiation therapy
PFS: Progression-free survival
OS: Overall survival
CBDCA: Carboplatin
PTX: Paclitaxel
NCCN: National Comprehensive Cancer Network
