# Supplementary material for: Squamous cell carcinoma of the anus successfully treated with multidisciplinary therapy for metachronous metastatic and local recurrences after DCF chemotherapy: a case report
Source: Surg Case Rep. 2024 Mar 25;10:71. doi: 10.1186/s40792-024-01873-2 (PMC10963677; doi:10.1186/s40792-024-01873-2)

(Additional images)

【MRI (T2WI, DWI) and PET-CT at the diagnosis (same time of Fig.1b)】

・T2WI


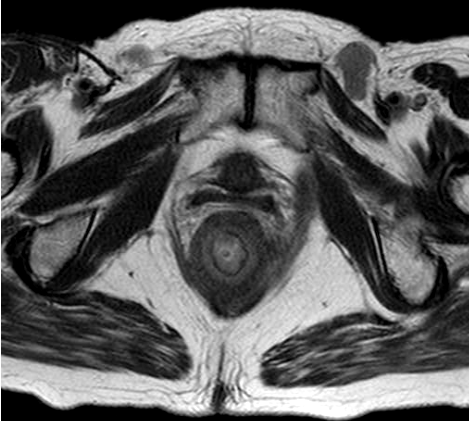


・DWI


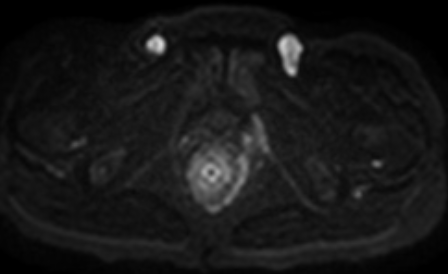


・PET-CT


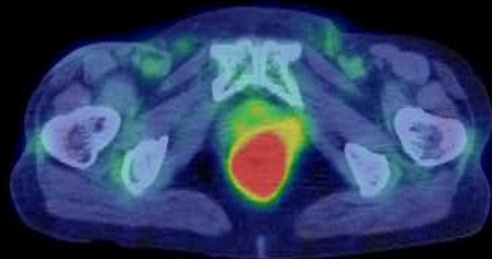


【CT and PET-CT at the time of para-aortic recurrence after DCF therapy (acquired at the same time as the images in Fig. 4a)】

・CT


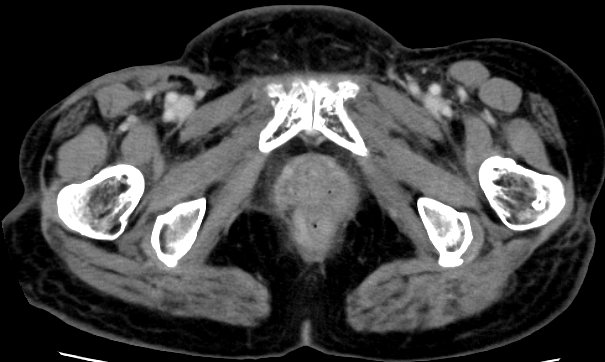


・PET-CT


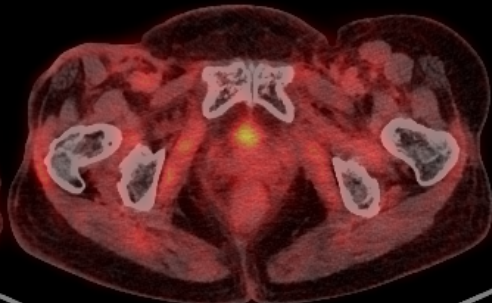


【CT, MRI(T2, DWI), and PET-CT at the time of local recurrence (the same time as that in Fig. 5)】

・CT


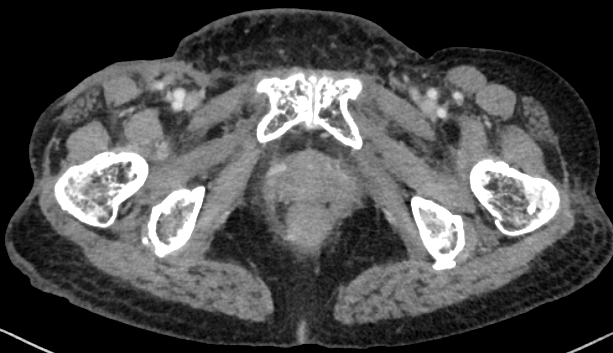


・T2WI


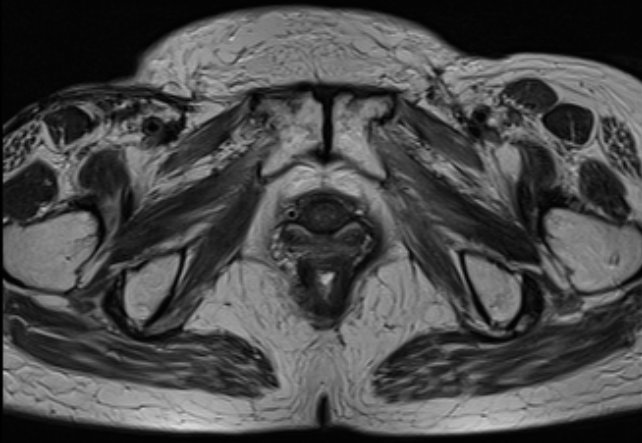


・DWI


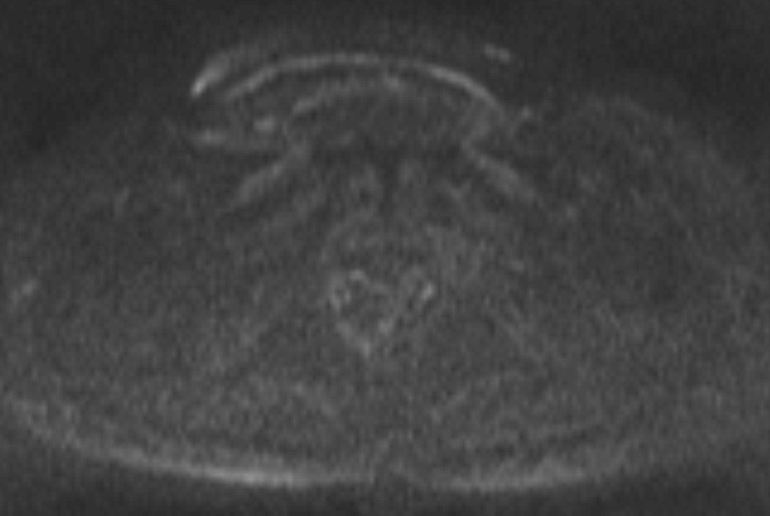


・PET-CT


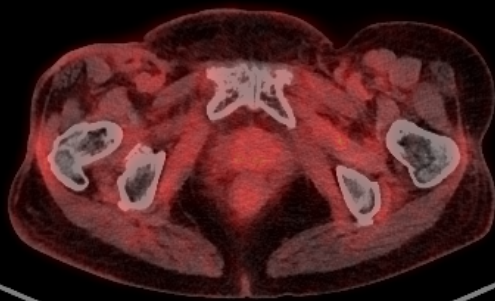

Supplement: Supplementary file 1 — Additional file 1. Additional images. [file 40792_2024_1873_MOESM1_ESM.docx]
